# Overfeeding Reduces Insulin Sensitivity and Increases Oxidative Stress, without Altering Markers of Mitochondrial Content and Function in Humans

**DOI:** 10.1371/journal.pone.0036320

**Published:** 2012-05-07

**Authors:** Dorit Samocha-Bonet, Lesley V. Campbell, Trevor A. Mori, Kevin D. Croft, Jerry R. Greenfield, Nigel Turner, Leonie K. Heilbronn

**Affiliations:** 1 Diabetes and Obesity Program, Garvan Institute of Medical Research, Darlinghurst, Australia; 2 Faculty of Medicine, University of New South Wales, Sydney, Australia; 3 School of Medicine and Pharmacology, University of Western Australia, Perth, Australia; 4 Department of Medicine, University of Adelaide, Adelaide, Australia; University of Cordoba, Spain

## Abstract

**Background:**

Mitochondrial dysfunction and increased oxidative stress are associated with obesity and type 2 diabetes. High fat feeding induces insulin resistance and increases skeletal muscle oxidative stress in rodents, but there is controversy as to whether skeletal muscle mitochondrial biogenesis and function is altered.

**Methodology and Principal Findings:**

Forty (37±2 y) non-obese (25.6±0.6 kg/m^2^) sedentary men (*n* = 20) and women (*n* = 20) were overfed (+1040±100 kcal/day, 46±1% of energy from fat) for 28 days. Hyperinsulinemic-euglycemic clamps were performed at baseline and day 28 of overfeeding and skeletal muscle biopsies taken at baseline, day 3 and day 28 of overfeeding in a sub cohort of 26 individuals (13 men and 13 women) that consented to having all 3 biopsies performed. Weight increased on average in the whole cohort by 0.6±0.1 and 2.7±0.3 kg at days 3 and 28, respectively (*P*<0.0001, without a significant difference in the response between men and women (*P* = 0.4). Glucose infusion rate during the hyperinsulinemic-euglycemic clamp decreased from 54.8±2.8 at baseline to 50.3±2.5 µmol/min/kg FFM at day 28 of overfeeding (*P* = 0.03) without a significant difference between men and women (*P* = 0.4). Skeletal muscle protein carbonyls and urinary F2-isoprostanes increased with overfeeding (*P*<0.05). Protein levels of muscle peroxisome proliferator-activated receptor gamma coactivator-1α (PGC1α) and subunits from complex I, II and V of the electron transport chain were increased at day 3 (all *P*<0.05) and returned to basal levels at day 28. No changes were detected in muscle citrate synthase activity or *ex vivo* CO_2_ production at either time point.

**Conclusions:**

Peripheral insulin resistance was induced by overfeeding, without reducing any of the markers of mitochondrial content that were examined. Oxidative stress was however increased, and may have contributed to the reduction in insulin sensitivity observed.

**Trial Registration:**

ClinicalTrials.gov NCT00562393

## Introduction

Obesity is closely linked with insulin resistance, and increasing evidence suggests that reactive oxygen species (ROS) generated in muscle mitochondria may impair insulin signalling in animal and cellular models [1], [2], [3]. These studies have also shown that over -expression of muscle-specific antioxidant enzymes [1], [2], or treatment with the mitochondrial superoxide dismutase (SOD) mimetics [2], [3] and mitochondria-specific free radical scavengers [1], protects rodents from developing insulin resistance following high fat overfeeding. Whether sustained high fat overfeeding will elevate these markers in non-obese humans is not yet clear, although a single high fat meal increases mitochondrial ROS emission in lean and obese humans [1]. Moreover, both systemic markers of oxidative stress and ROS production in skeletal muscle mitochondria are reported to be elevated in human obesity [1], [4], [5].

A reduction in skeletal muscle mitochondrial number and/or maximal oxidative capacity is also reported in human obesity, aging and type 2 diabetes [6] and is postulated to be causal in the development of obesity-associated insulin resistance. The mitochondrial dysfunction hypothesis of insulin resistance has arisen mainly from studies showing reduced expression of genes involved in mitochondrial biogenesis or reduced ATP production in healthy relatives of type 2 diabetes individuals [6]. Reduced expression of genes involved in mitochondrial biogenesis is also observed following isocaloric high fat diet, or following prolonged lipid infusion with the parallel induction of peripheral insulin resistance in healthy humans [7], [8], [9]. However, other studies have shown that mitochondrial dysfunction is not a prerequisite for insulin resistance in humans [5], [10], [11], [12]. Rodents that are fed a high fat diet for 4–20 weeks have increases in the more functional measures of skeletal muscle oxidative capacity, despite developing insulin resistance and diabetes [13], [14], [15]. Together, these findings challenge the role of mitochondrial dysfunction as a primary factor in the development of insulin resistance.

We, and others, have previously shown that short term overfeeding decreases the glucose infusion rate necessary to maintain euglycemia during a hyperinsulinemic-euglycemic clamp [16], [17]. In this study, we focused on factors in skeletal muscle that may contribute to the insulin resistance that was observed during overfeeding. The specific aims were to determine the effects of 3 and 28 days of overfeeding on (i) skeletal muscle markers of oxidative stress, and (ii) mitochondrial content and function. We hypothesized that overfeeding would increase oxidative stress and this would be associated with a reduction in markers of mitochondrial content and function.

## Methods

This study was conducted according to the principles expressed in the declaration of Helsinki. The study was approved by the Human Research and Ethics Committee at St Vincent’s Hospital, Sydney. All participants provided written informed consent for the collection of samples and subsequent analysis. The protocol for this study and supporting CONSORT checklist are available as supporting information (Protocol S1 and Checklist S1).

### Participants

**Figure 1:**
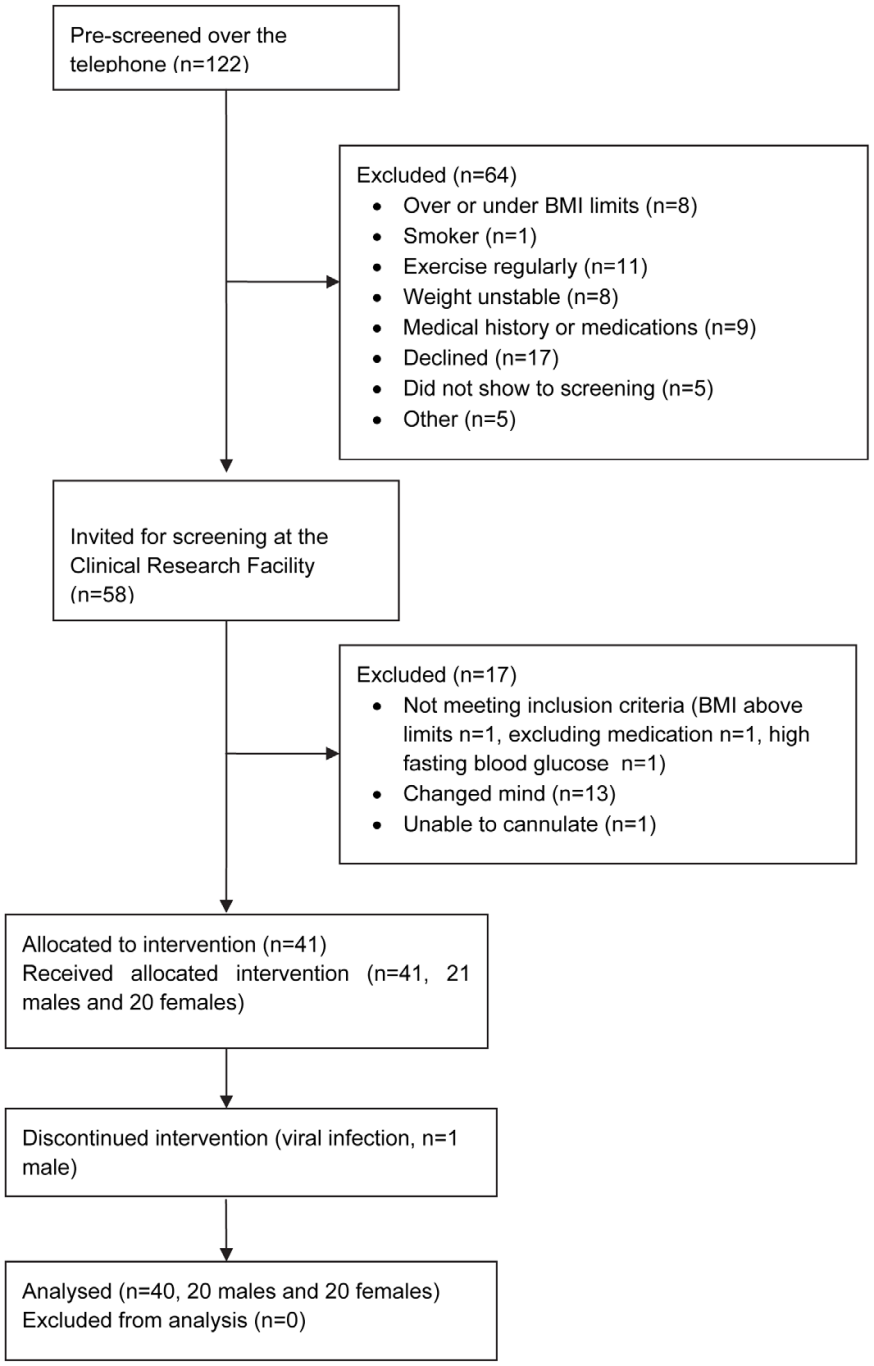
Flow of participants in the study.

Of the 122 individuals pre-screened for the study over the telephone, 64 were excluded (Figure 1). Fifty eight individuals were screened at the Clinical Research Facility of which 17 were excluded (3 did not meet the inclusion criteria, 13 changed their mind after the study was explained and 1 due to difficulty in cannulating; Figure 1). Forty one participants were enrolled (36 Caucasian, 5 Asian) and 1 individual withdrew due to a viral infection. Forty individuals completed the study (20 men and 20 women; 37±2 years). Participants were either without a family history of diabetes (FH- *n* = 23) or FH+ (at least one first-degree relative with type 2 diabetes, *n* = 17), and 5 women were post-menopausal. Participants were excluded if they smoked, had a BMI >33 kg/m^2^, weight change >2 kg in the preceding 6 months, exercised >60 min/week, were treated with medications known to affect insulin sensitivity, took nutritional supplements and/or had a personal history of diabetes or cardiovascular disease. All participants were recruited and followed up between 2007 and 2009 as reported previously [17], [18].

### Diets

Study timeline and diet regimen were described in detail previously [17]. Briefly, baseline diet was provided from day −3 to day −1 at calculated energy requirements with a nutrient composition of 30% fat, 15% protein and 55% carbohydrate. The overfeeding diet (day 0−day 28) was calculated as 1250 kcal/ d above baseline energy requirements with a nutrient composition of 45% fat, 15% protein and 40% carbohydrate. Overfeeding was achieved by supplementing the baseline diet with energy-dense snacks that were provided to study participants. Specifically, 3 high energy-high fat snacks per day, each providing ∼250 kcal (e.g. potato crisps, chocolate bars, cheesecake) and a liquid oil-based supplement (Benecalorie®, Novartis, Basel, Switzerland, 330 kcal) mixed in a dairy dessert (∼200 kcal). Participants were required to fill out checklists daily reporting which snacks were consumed and to complete diet diaries (3 days) before commencing the study and twice during overfeeding (*n* = 32).

### Metabolic Testing

Metabolic tests used in this study included hyperinsulinemic-euglycemic (60 mU/m^2^/min, 2-h) clamp combined with indirect calorimetry and fat mass and fat-free mass assessment by DXA at day 0 and day 28 (as described previously [17]). A vastus lateralis muscle biopsy was performed, as previously described [19]. Briefly, the muscle samples (∼200 mg) were rinsed in ice-cold saline, blotted for blood, and any visible fat removed. Samples were then snap-frozen in liquid nitrogen, within 90 seconds of collection.

### Biochemical Analysis

Blood measurements were performed on the whole cohort (*n* = 40). Blood glucose and plasma lactate were assessed by YSI 2300 (YSI Life Sciences) and serum insulin by RIA (Linco Research, St Charles). Serum non esterified fatty acids (NEFA) were analyzed by an enzymatic colorimetry assay (Wako, Osaka, Japan). The capacity of the serum to inhibit the oxidation of 2,2′-Azino-di-3-ethylbenz-thiazoline sulphonate (ABTS) was tested by a colorimetric kit (Calbiochem, EMD Biosciences, Inc. CA). Urinary-F2-isoprostane was analyzed using gas chromatography-mass spectrometry in spot urine samples that were centrifuged at 4°C, snap-frozen with butylated hydroxytoluene (BHT, 0.005%) and stored at −80°C. The results were normalized to creatinine content [20].

### Skeletal Muscle Processing

Muscle measurements were performed on the sub-cohort (*n* = 26) who consented to having all 3 biopsies (baseline, 3 and 28 days), unless otherwise stated. Muscle was homogenized 1∶14 (wt/vol) in 50 mmol/L Tris-HCl, 1 mmol/L EDTA and 0.1% Triton X-100, pH 7.2, using a Polytron instrument (Kinematica, Littau-Lucerne, Switzerland), unless otherwise stated.

### Immunoblotting

Frozen muscle samples were resuspended in radioimmunoprecipitation buffer supplemented with protease and phosphatase inhibitors as described [15], [19]. Equal amounts of tissue lysates (10–20 µg protein) were resolved by SDS-PAGE (Criterion 4–12% Resolving Gel, Bio-Rad, US) and immunoblotted with antibodies against peroxisome proliferator-activated receptor gamma coactivator 1 (PGC1α, Chemicon International, Temecula, CA), muscle carnitine palmitoyltransferase (CPT-1, Alpha Diagnostic International, San Antonio, TX), uncoupling protein 3 (UCP3, affinity Bioreagents, Golden, CO), Mn-SOD (Santa Cruz Biotechnology, CA) and an antibody cocktail that recognizes several subunits of the mitochondrial respiratory chain (MS601, Mito Sciences, Eugene, OR). Beta-actin (Santa Cruz Biotechnology, CA) was used to verify that equal amounts of proteins were loaded. Immunolabelled bands were quantified by densitometry.

### Enzymes Activity Measurements

Muscle lysates were subjected to 3 freeze-thaw cycles. Citrate synthase, β-hydroxyacyl CoA dehydrogenase, hexokinase and phosphofructokinase were determined at 30°C, as described [19], using a Spectra max 250 microplate spectrophotometer (Molecular Devices, Sunnyvale, CA).

### Skeletal Muscle Palmitate Oxidation

Palmitate oxidation was measured at baseline and day 28 only, as previously described [15] with modified concentration of the reaction mixture and substrates. Briefly, freshly collected muscle (∼50 mg) was homogenized in 19 vol of ice-cold 250 mM sucrose, 10 mmol/L Tris-HCL, and 1 mmol/L EDTA, pH 7.4 buffer and 100 µl of muscle homogenate was incubated with 400 µl reaction mixture. Final concentrations of the reaction mixture were (in mmol/L): 125 sucrose, 2.5 ATP, 12.5 Tris-HCL, 6.25 KH_2_PO_4_, 1.25 MgCl_2_, 100 KCl, 0.25 EDTA, 1.25 dithiotreitol, 2.5 malate, 0.25 palmitate and 0.38% fatty acid-free BSA. Substrate was 0.2 mmol/L [1-^14^C] palmitate (0.1 µCi), 2.5 mmol/L _L_-carnitine and 0.063 mmol/L CoA.

### Skeletal Muscle Protein Carbonylation

Skeletal muscle protein carbonylation was measured by the Oxyblot Protein Oxidation Detection Kit (Millipore, Canada) according to the manufacturer’s instructions. This analysis was performed last on all subjects with sufficient muscle lysate remaining (*n* = 18).

### Statistical Analysis

Data presented as mean ±SEM. Repeated measures ANOVA was used to detect the effect of overfeeding on the outcome measures on the whole cohort and between men and women, unless otherwise stated. Skeletal muscle protein activity and level were expressed relative to baseline and statistical significance was tested by t-test. Insulin data were log_10_-transformed. Pearson’s correlations were performed. SPSS Statistics 19 (Chicago, IL) was used, without adjustment for multiplicity. Any missing data was removed from the analysis, and not carried forward or replaced. Sample size requirements were calculated for primary endpoint Δ (glucose infusion rate/kg FFM) based on previous data [19] using 2 sided within subject contrasts with α<0.05 and statistical power 1-β>0.8.

## Results

In this cohort, we have previously reported that individuals with a family history of type 2 diabetes (FH+) exhibited similar insulin sensitivity, fasting blood glucose and plasma insulin at baseline to age, and fatness matched individuals without a family history of type 2 diabetes (FH-) [17]. In response to overfeeding, FH+ gained more weight and had greater increases in fasting insulin and glucose, although we did not detect a difference between groups for the change in insulin sensitivity by clamp [17]. Here, we report that none of the markers of oxidative stress or skeletal muscle mitochondrial content measured were different between groups at baseline or in response to overfeeding, and thus groups were combined for the statistical analyses below.

### Anthropometric and Metabolic Responses to Overfeeding

Overfeeding led to an average weight gain of 0.6±0.1 kg at day 3 and 2.7±0.3 kg at day 28 in the whole cohort and this response was not significantly different between men and women (Table 1). Body fat also increased significantly at day 28, without a significant difference between men and women (Table 1). The increases in total energy, total fat, saturated, monounsaturated and polyunsaturated fat, protein and carbohydrate intake from baseline to overfeeding were not different between men and women (*P*>0.2 for all). The mean energy, macronutrient composition and their contribution to total energy of baseline and overfeeding diets is provided for the whole cohort in Table 2. Fasting glucose and insulin increased significantly in the whole cohort (*P* = 0.001 and *P*<0.0001 for glucose and insulin, respectively), and the response was not significantly different between men and women (Table 1). Likewise, insulin resistance by the homeostasis model of assessment of insulin resistance (HOMA-IR) increased significantly and glucose infusion rate during the hyperinsulinemic-euglycemic clamp decreased significantly with overfeeding and the response was not significantly different between men and women (Table 1).

**Table 1:** Anthropometric and metabolic responses to overfeeding in the whole cohort and in men and women.

	Whole cohort (*n* = 40)			Men (*n* = 20)			Women (*n* = 20)			P	
	Baseline	3-days	28-days	Baseline	3-days	28-days	Baseline	3-days	28-days	Time	Group
Weight(kg)	75.3±1.9	75.9±1.9**	78.1±1.9**	81.3±2.3	81.8±2.3**	84.3±2.3**	69.4±2.3	70.0±2.3**	71.9±2.4**	0.0001	0.4
BMI (kg/m_2_)	25.6±0.6	25.8±3.6**	26.6±3.6**	25.4±0.7	25.6±0.7**	26.4±0.7**	25.8±0.9	26.0±0.9**	26.7±1.0**	0.0001	0.8
Total body fat (%)	34±1	–	35±1**	29±2	–	30±2**	40±2	–	41±2**	0.0001	0.7
Glucose (mmol/L)	4.5±0.06	4.7±0.06**	4.6±0.05*	4.60±0.07	4.80±0.08*	4.60±0.05	4.40±0.08	4.60±0.07**	4.60±0.09**	0.001	0.2
Fasting insulin (pmol/L)	65.5±3.3	80.3±4.5**	77.2±3.6**	65.6±3.6	79.3±6.6**	77.6±5.5*	68.3±5.4	81.2±6.3*	76.9±4.9	0.0001	0.6
HOMA-IR	1.8±0.1	2.3±0.1**	2.2±0.1**	1.8±0.1	2.4±0.2**	2.2±0.2	1.8±0.1	2.3±0.2**	2.2±0.2*	0.0001	0.8
GIR (µmol/Kg FFM/min)	54.8±2.8	–	50.3±2.5*	53.6±4.7	–	47.5±4.0	56.0±3.2	–	53.2±2.8	0.03	0.4
NEFA(mmol/L)	0.30±0.02	0.19±0.02**	0.30±0.02	0.21±0.02	0.14±0.02**	0.20±0.02	0.38±0.03	0.23±0.03**	0.37±0.03	0.0001	0.05
Lactate (mmol/L)	0.68±0.04	0.78±0.04*	0.75±0.04	0.76±0.07	0.87±0.05	0.90±0.05	0.59±0.05	0.69±0.06	0.62±0.05	0.03	0.4
Respiratory quotient	0.81±0.01	0.85±0.01**	0.82±0.01	0.82±0.01	0.87±0.01**	0.84±0.01	0.80±0.01	0.84±0.01**	0.80±0.01	0.0001	0.5

Data presented as mean ±SEM for the whole cohort (*n* = 40) and for men (*n* = 20) and women (*n* = 20).

Significance of the repeated measure ANOVA with time and between groups is given.

Difference from baseline (by paired t-test).

* *P*<0.05,

** *P*<0.01.

**Table 2:** Composition of the diet at baseline and during overfeeding.

	Baseline	Overfeeding
Energy (kcal/d)	1980±110	3100±140
Carbohydrate (g/d)	210±10	280±20
Carbohydrate (% of energy)	45±1	38±1
Alcohol (g/d)	6±2	5±1
Protein (g/d)	90±5	120±5
Protein (% of energy)	19±1	16±1
Fat (g/d)	77±5	156±6
Fat (% of energy)	34±1	45±1
Saturated (g/d)	29±2	54±3
Saturated (% of fat)	41±1	37±1
Polyunsaturated (g/d)	13±1	17±1
Polyunsaturated (% of fat)	18±1	12±1
Monounsaturated (g/d)	28±2	74±3
Monounsaturated (% of fat)	40±1	50±1

Data presented as mean ±SEM and based on 3 day diet diaries filled before commencement of the study and twice during the overfeeding intervention (*n* = 32).

### Markers of Oxidative Stress in Response to Overfeeding

**Figure 2:**
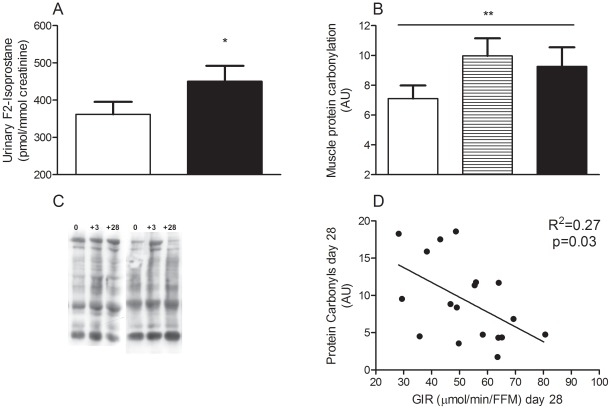
Urinary F2-isoprostane content and skeletal muscle protein carbonyls in response to overfeeding. Urine F_2_-isoprostane (A), skeletal muscle protein carbonyls quantification (B) and representative blots (C) and the association between protein carbonyls and peripheral insulin resistance at end of overfeeding (D). Data are expressed as mean±SEM at baseline (white), day 3 (striped) and day 28 (black) of overfeeding. Difference by RM-ANOVA ^*^ *P*<0.05, ^**^ *P* = 0.01, urinary F2-isoprostanes data is based on *n* = 37 (18 men and 17 women) and skeletal muscle protein carbonyl on *n* = 18 (8 men and 10 women).

**Figure 3:**
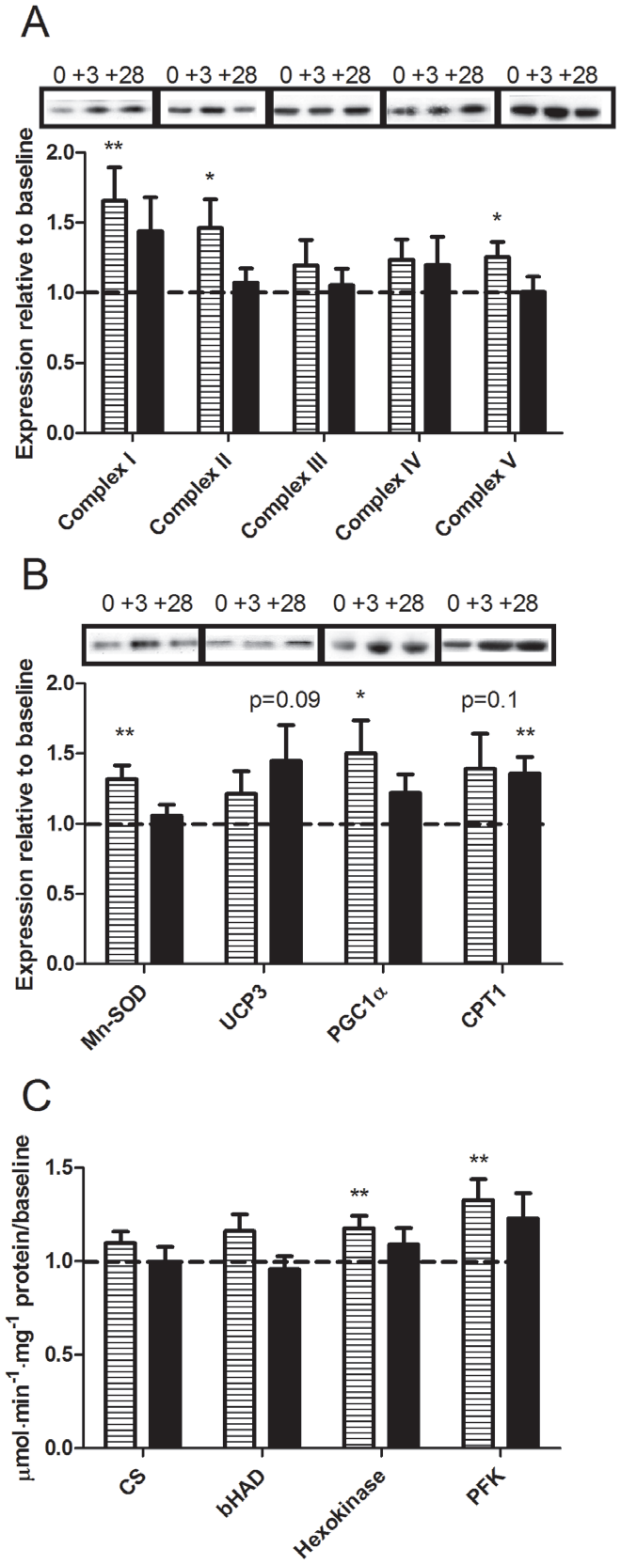
Skeletal muscle protein expression and enzyme activities in response to overfeeding. Skeletal muscle complexes of the electron transport chain (a), Mn-superoxide dismutase (SOD), uncoupling protein-3 (UCP3), PPAR-coactivator 1α (PGC1α), and carnitine palmitoyltransferase (CPT1b) proteins and representative samples of the Western blots (b) and skeletal muscle citrate synthase (CS), hydroxyacyl-CoA dehydrogenase (βHAD), hexokinase and phosphofructokinase (PFK) activities (c). Data expressed relative to baseline at day 3 (striped) and day 28 (black) of overfeeding (*n* = 26; 13 men and 13 women). Difference by one-way t-test ^*^ *P*<0.05, ^**^ *P*<0.01.

Urinary F2-isoprostane content, a marker of *in vivo* oxidative stress [21] increased at day 28 of overfeeding in the whole cohort (*P* = 0.03, Figure 2A) and this response was not different between men and women (data not shown). While baseline skeletal muscle protein carbonyls were not correlated with baseline insulin resistance, protein carbonyl level increased at both 3 and 28 days (*P* = 0.01, Figure 2B quantification and 2C representative blots) and was correlated with peripheral insulin resistance at day 28 (Figure 2D). Serum antioxidative capacity was unchanged (1.1±0.1, 1.0±0.1 and 1.0±0.1 mM at baseline, 3 and 28 days, respectively *P* = 0.2) and there was no difference in response between men and women (data not shown). Protein content of complex I, but not complex III, of the mitochondrial electron transport chain was elevated at day 3 (Figure 3A), and there was no difference in response between men and women (data not shown). However, despite continued overfeeding this was not sustained at day 28. Similarly, a transient increase in MnSOD was observed at day 3 (Figure 3B). Uncoupling protein 3 (UCP3) tended to increase at day 28 (Figure 3B, *P* = 0.09). No relationships were observed between the change in insulin resistance and the change in F2-isoprostanes and protein carbonylation with overfeeding or between UCP3, complex I or MnSOD and markers of oxidative stress with overfeeding.

### Markers of Lipid Metabolism and Mitochondrial Function in Response to Overfeeding

Protein levels of complexes I, II and V of the mitochondrial electron transport chain (Figure 3A) and PGC1α (Figure 3B) transiently increased at day 3, suggesting an increase in mitochondrial biogenesis, and there was no difference in response between men and women (data not shown). However, more functional markers of mitochondrial oxidative capacity and content, namely palmitate oxidation rate by muscle homogenates *ex vivo* and citrate synthase activity, were not altered by overfeeding (23±4 *vs.* 24±4 µmol/g/min CO_2_ at baseline and day 28, *P* = 0.9 and Figure 3C, respectively). Muscle carnitine palmitoyltransferase (CPT)1b protein level tended to increase at day 3 and this was statistically significant at day 28 (Figure 3B), reflecting an increase in fatty acid entry into the mitochondria. However, β-hydroxyacyl CoA dehydrogenase (βHAD) activity was unchanged (Figure 3C). The activity of the glycolytic enzymes hexokinase and phosphofructokinase (PFK) were increased at day 3 (Figure 3C) and this was consistent with the transient increase in the respiratory quotient (RQ) and fasting plasma lactate and the decrease in plasma NEFA at that time point (Table 1). Notably, while NEFA concentrations were significantly decreased in both men and women at day 3, the decrease was greater in women compared with men (Table 1).

## Discussion

Short term overfeeding reduces insulin sensitivity in healthy non-obese individuals [16], [17], however the mechanisms underlying this are unclear. In this study, we report that whilst the reduction in insulin sensitivity following overfeeding was modest, it occurred without a reduction in any of the markers of mitochondrial content and function examined. However, we observed that systemic and skeletal muscle markers of oxidative stress were increased, and therefore may have contributed to the insulin resistance observed.

The role of ROS in mediating insulin resistance is debated [22]. Chronic ROS production by skeletal muscle mitochondria can inhibit insulin action but paradoxically, acute increases in ROS through NADPH-oxidase (NOX) are required for normal intracellular signalling [23]. Increased ROS production is common to different models of cellular insulin resistance, including those induced by TNF-α, insulin and palmitate treatments [2], [3]. Moreover, mitochondria-targeted antioxidant treatment partially preserves insulin sensitivity both *in vivo* [1], [2], [3] and *in vitro* [2], [3]. In the present study, we observed that both urinary F2-isoprostane and skeletal muscle protein carbonyls were increased, with the latter increased as early as 3 days of overfeeding. This finding suggests that increased oxidative stress may be an early event during over-nutrition in humans. Protein carbonylation is a non-reversible modification by highly reactive aldehydes, by-products of lipid peroxidation that cause loss of function or trigger degradation of proteins with a cysteine, histidine or lysine side chain, typically enzymes [24]. Carbonylated proteins, including the antioxidants thioredoxin, thioredoxin reductase, glutathione peroxidase, fatty acid binding protein and cytosolic and mitochondrial NADP^+^-dependent isocitrate dehydrogenase isoforms, were 2–3 fold higher in adipose tissue collected from high fat-high sucrose fed mice compared to chow fed mice [24]. We speculate that antioxidants and enzymes involved in oxidative stress and/or insulin action in skeletal muscle may also be potential targets for carbonylation and degradation during the overfeeding diet. Also, in the postprandial state, fat and carbohydrate have a differential effect on the oxidative stress response [25]. Importantly, participants in the present study were placed on the same snacks, rich in both sugar and fat, to increase their energy intake and thus we cannot differentiate the effect of particular macronutrients on the outcomes. The two principal sites of superoxide generation in mitochondria are complexes I and III of the electron transport chain. In this study, we observed an increase in protein content of complex I, but not complex III, at day 3 of overfeeding. However, despite continuous overfeeding and potentially increased availability of reducing equivalents in the mitochondria, this was not sustained. Superoxide leaking from the mitochondrial complexes is dismutated rapidly into hydrogen peroxide by MnSOD and Cu/ZnSOD in the mitochondrial matrix and the inter-membrane space, respectively. Consistent with this, it has previously been shown that MnSOD transgenic mice are partially protected from high fat feeding-induced insulin resistance [2]. In the present study, we observed that MnSOD was increased transiently, possibly in an attempt to limit oxidative damage. We speculate that the lack of a sustained induction of the anti-oxidative systems, including MnSOD and UCP3 may have contributed to the increase in oxidative stress that was observed following overfeeding in this study. Previous studies have shown that high fat diet increased UCP3 protein in rodent mitochondria [26] and isocaloric 65% fat diet increased UCP3 mRNA expression in lean, but not obese humans [27]. However, to our knowledge protein content of UCP3 during overfeeding has not previously been investigated.

Pre-diabetes and type 2 diabetes are characterized by reduced expression and protein levels of PGC1α, a master metabolic regulator of mitochondrial biogenesis [6]. However, it is unclear whether this is a cause or consequence of insulin resistance. In the present study, the protein levels of PGC1α and the complexes of the mitochondrial electron transport chain were increased at day 3, but these returned to basal at day 28. This finding is supported by studies in rodents showing that a high fat diet for 5–20 weeks increased PGC1α and the activity and expression of proteins involved in skeletal muscle oxidative capacity [9], [13], [14], [15], [28], [29] in concert with induction of glucose intolerance and/or insulin resistance [13], [15]. However, other studies in rodents [8], [9], [30] and humans [8], [31] have reported that high fat diet induced a coordinated downregulation in markers of mitochondrial biogenesis. Notably, studies in humans were limited to the assessment of mRNA expression [8], [31], which are not necessarily reflective of protein levels. In this study, we did not detect any change in palmitate oxidation and citrate synthase activity with overfeeding, and *in vivo* mitochondrial capacity was not assessed.

We chose to focus on skeletal muscle as the potential mediator of the reduction in peripheral insulin sensitivity that was observed. A potential limitation of this study is that we did not perform 2-step clamps with tracers to assess the effects of overfeeding on hepatic insulin sensitivity. Previous studies have reported hepatic insulin resistance in response to high fat overfeeding in rodents [32] and humans [33]. The increase in HOMA-IR that was observed in this study is supportive of this notion. Moreover, timeline studies performed in rodents have shown that whilst hepatic insulin resistance is observed within 3 days of high fat diet, this is followed by insulin resistance in muscle only at 3 weeks [32]. We highlight that the insulin dose in the hyperinsulinemic clamp that was chosen here will have completely suppressed hepatic glucose production, and that the muscle is the major site of glucose uptake at this dose in both rodents and humans. Interestingly, we also observed that despite a doubling in dietary fat intake, there was little evidence of an increase in fatty acid oxidation in muscle, besides an increase in CTP1b protein, the rate limiting step for entry of long chain fatty acyl-CoA into mitochondria. Rather our data support previous human studies [34] and suggests that the immediate response to overfeeding an energy dense diet is to suppress adipose tissue lipolysis and oxidize glucose, at least in the fasting state, with transient decreases in NEFA and increases in lactate, skeletal muscle glycolytic enzyme activities and whole body glucose oxidation. Few gender differences were detected in this study, but, we recognize that the cohort size may have been too small to detect some effects between men and women, especially for endpoints in muscle in which a reduced cohort was available for analysis.

In conclusion, peripheral insulin sensitivity decreased without reducing the markers of mitochondrial content examined. Oxidative stress was however increased, and may have contributed to the decrease in insulin sensitivity that was observed. However, conclusions regarding the temporal sequence of events and whether these effects are a consequence of overfeeding or of weight gain cannot be drawn in the present study. We speculate that with prolonged overfeeding and sustained increases in ROS, oxidative stress-sensitive mitochondrial proteins involved in metabolism may be impaired, resulting in mitochondrial dysfunction and skeletal muscle lipid accumulation that generally characterize obesity, insulin resistance and type 2 diabetes.

## Supporting Information

Checklist S1 **CONSORT Checklist.**
(DOC)

Protocol S1 **Trial Protocol.**
(DOC)
