# Supplementary material for: Overfeeding Reduces Insulin Sensitivity and Increases Oxidative Stress, without Altering Markers of Mitochondrial Content and Function in Humans
Source: PLoS One. 2012 May 7;7(5):e36320. doi: 10.1371/journal.pone.0036320 (PMC3346759; doi:10.1371/journal.pone.0036320)
Supplement: Protocol S1 — Trial Protocol. (DOC) [file pone.0036320.s002.doc]

**ST VINCENT’S HOSPITAL/University of New South Wales**

**ETHICS APPLICATION FORM FOR RESEARCH INVOLVING HUMANS**

**Effects of Excess Energy Intake on Metabolic Health**

Office use only

Project Number:

Date Reviewed:

Date Approved:

**SECTION 1: ADMINISTRATION AND SUBMISSION TO OTHER INSTITUTIONAL ETHICS COMMITTEES (IECs)**

**1.1 (a) Full project title**

Effects of EXCESS Energy Intake on Metabolic Health.

**(b) Short name by which the project will be known (if appropriate)**

EXCESS

**(c) Name of Chief Investigator**

Lesley Campbell

**(d) Provide a brief lay description of the project (in less than 100 words).**

The prevalence of obesity is rapidly increasing in Australia is estimated to cost >$3.6 billion per year. In this study, we will investigate the effects of over-nutrition in lean individuals, overweight individuals and individuals who have a strong family history of diabetes. The aims is to distinguish physiological and endocrine characteristics of individuals who store more fat in response to overfeeding and identify defects in machinery involved in fat oxidation in skeletal muscle. We will re-examine overweight individuals again after 10% weight loss. This study will determine whether overweight and lean subjects behave similarly when faced with over-nutrition, establish quantifiable markers to distinguish those at risk of insulin resistance and identify new targets for intervention.

**1.2 Indicate the institution that you consider to be the primary site for this research project.**

Garvan Institute

**1.3 List the following details of the Chief Investigator/Supervisor, any Co-Investigator,**

**Associate Investigator and Student. (If necessary, insert extra pages to follow this.)**

**Chief Investigator/Supervisor**

| **name** | **Lesley Campbell** |
| --- | --- |
| title | Prof |
| qualifications | MBBS, PHD |
| positions held | Director, Diabetes Centre |
| full mailing address | 372 Victoria St, Darlinghurst, 2010 |
| telephone number | (02) 8283 2622 |
| fax number | (02) 9295 8201 |
| e-mail address | [l.campbell@garvan.org.au](mailto:l.campbell@garvan.org.au) |

**Co-Investigator**

| **Name** | **Leonie Heilbronn** |  |
| --- | --- | --- |
| Title | Dr |  |
| full mailing address | 384 Victoria St, Darlinghurst, NSW 2010 |  |
| telephone number | (02) 9295 8309 |  |
| fax number | (02) 9295 8201 |  |
| e-mail address | [l.heilbronn@garvan.org.au](mailto:l.heilbronn@garvan.org.au) |  |

**Students:**

| **name** | **Alexander Viardot, MBBS** |
| --- | --- |
| title | Dr |
| full mailing address | 384 Victoria St, Darlinghurst, 2010 |
| telephone number | 9295 8310 |
| fax number | 9295 8201 |
| e-mail address | [a.viardot@garvan.org.au](mailto:a.viardot@garvan.org.au) |

| **name** | **Kerry-Lee Milner, MBBS** |
| --- | --- |
| title | Dr |
| full mailing address | 384 Victoria St, Darlinghurst, 2010 |
| telephone number | 9295 8215 |
| fax number | 9295 8201 |
| e-mail address | [k.milner@garvan.org.au](mailto:k.milner@garvan.org.au) |

1.4 (a) Indicate the proposed date of commencement of the project. Researchers are reminded that projects may not commence without the written approval of the Institutional Ethics Committee (IEC).

Jan 2007

1. **Indicate the proposed duration of the project.**

2-years

**1.5 Indicate the location at which the research will be undertaken.**

Garvan Institute

| **1.6** | **(a)** | **Is this submission being made as part of an application for research funding?** | **No** | **Yes** |
| --- | --- | --- | --- | --- |

If you answered **YES**, list the funding bodies to which you have submitted, or intend to submit, this project.

An NHMRC Project Grant has been awarded for this project.

**(b) If the title of the project submitted for funding is different from that listed**

**under Question 1.1, state it below.**

Short-term effects of over-feeding on metabolic risk in humans

| **1.7** | **Has this project been submitted to any other IEC(s)?** |  |  |
| --- | --- | --- | --- |
|  |  | **No** | **Yes** |

If you answered **YES**, give the name of the IEC(s), and indicate the status of the application at each (i.e., submitted, approved, deferred or rejected).

Attach copies of the correspondence with each of the other IEC(s).

##### SECTION 2: NATURE OF RESEARCH INCLUDING RISKS

**2.1 The nature of this project is most appropriately described as :**

|  | (a) | A clinical trial of drug(s) or device(s |  |  |
| --- | --- | --- | --- | --- |
|  |  |  | No | Yes |

|  |  | (i) under the Clinical Trial Notification Scheme (CTN) |  |  |
| --- | --- | --- | --- | --- |
|  |  |  | No | Yes |

|  |  | (ii) under the Clinical Trial Exemption Scheme (CTX ) |  |  |
| --- | --- | --- | --- | --- |
|  |  |  | No | Yes |

|  |  | (ii) under the Clinical Trial Exemption Scheme (CTX ) |  |  |
| --- | --- | --- | --- | --- |
|  |  |  | No | Yes |

|  |  | (iii) using only approved drug(s)/device(s) in accordance with Therapeutic Goods Administration Approved Product Information | No | Yes |
| --- | --- | --- | --- | --- |

|  | (b) | Human physiology research |  |  |
| --- | --- | --- | --- | --- |
|  |  |  | No | Yes |

|  | (c) | Human tissue research |  |  |
| --- | --- | --- | --- | --- |
|  |  |  | No | Yes |

|  | (d) | Psychiatry/clinical psychology research |  |  |
| --- | --- | --- | --- | --- |
|  |  |  | No | Yes |

|  | (e) | Behavioural research |  |  |
| --- | --- | --- | --- | --- |
|  |  |  | No | Yes |

|  | (f) | Biomechanical research |  |  |
| --- | --- | --- | --- | --- |
|  |  |  | No | Yes |

|  | (g) | Research using a questionnaire only |  |  |
| --- | --- | --- | --- | --- |
|  |  |  | No | Yes |

|  | (h) | Research using qualitative methods |  |  |
| --- | --- | --- | --- | --- |
|  |  |  | No | Yes |

|  | (i) | Other (indicate the nature of the research below) |  |  |
| --- | --- | --- | --- | --- |
|  |  |  | No | Yes |

| **2.2** | **(a)** | **Does the protocol require any physically invasive, or potentially harmful procedures (e.g. drug administration, needle insertion, rectal probe, pharyngeal foreign body, electromagnetic stimulation)?** | **No** | **Yes** |
| --- | --- | --- | --- | --- |

If you answered **YES**, state the nature of the procedures, all the risks involved and, if possible, at what rate these risks are expected to occur. (All this information must be included in the Subject Information Statement.)

The procedures listed below have previously been approved by the SVH Human Research Ethics Committee and have been conducted within our group at the Garvan Institute for many years

Hyperinsulinaemic euglycaemic Clamp and Metabolic rate

This procedure measures whole body insulin sensitivity and secretion. Subjects will fast for 12-h overnight. Two intravenous cannulae will be inserted. One will be inserted into an antecubital vein and the other inserted in a retrograde manner into a contralateral hand vein. This hand will be thermostatically heated in an electric blanket to 50ºC to provide arterialised venous blood for sampling. Baseline bloods will be drawn for measurement of lipid profiles and glucoregulatory hormones. Insulin sensitivity will be assessed using a constant infusion of human insulin (60mu/m^2^/min) for 120 minutes. Blood glucose will be maintained at 5mmol/L with a supplemental infusion of D-glucose. The amount of glucose required to maintain normoglycaemia is proportional to insulin sensitivity. Metabolic rate by indirect calorimetry (DeltraTrac, Sensor Medics) will be measured immediately prior to and for the last 30 minutes of the clamp. This involves a ventilated transparent plastic hood connected to the device placed over the head of the subject. Calculations of O_2_ consumption and CO_2_ production will be made from continuous measurements of CO_2_ and O_2_ exchange. Subjects will be asked to remain motionless and awake during the test. The total time for these procedures is 3 hours.

DEXA: This will be performed at St Vincent’s Hospital. The protocol requires subjects to lie on a table wearing a hospital gown and no metal containing objects, while the scanner emitting low energy X-rays, and a detector passes along the body. The scan takes less than 5 minutes and the radiation dose is <5 microseiverts, equal to about 12-h of background radiation.

Fat biopsy: A subcutaneous abdominal adipose biopsy will be performed using the technique ot Bergstrom: after cleansing the skin on the abdomen with povidone-iodine solution, and placing a sterile drape, anesthesia is administered with 5mL of a 50% -50% mixture of lidocaine and bupivacaine. A 0.75-cm incision is made in the skin (#11 scalpel) and a 4mm Bergstrom needle inserted to collect under aspiration approximately 250-350mg of adipose tissue. Two or three passes will be used to obtain approximately 700mg of adipose tissue. The sample is washed in sterile PBS and snap frozen in liquid nitrogen. Upon completion of the biopsy, the incision is closed with a sterile bandage, and a sterile dressing applied.

Muscle biopsy: A Vastus Lateralis muscle biopsy will be performed using the technique of Bergstrom: after cleansing the skin with povidone-iodine solution, the skin, adipose tissue and skeletal muscle fascia are anesthetized using 5-10mL of a 50%/50% mixture of bupivicaine and lidocaine. The skin is incised (0.75cm) with a #11 scalpel. The fascia fibers are separated with the blunt edge of the scalpel and the Bergstrom needle (4mm) inserted into the vastus lateralis. After suction is applied and ~100mg of tissue is cut and removed. Three passes will be used to obtain ~250mg. Pressure is applied and the skin is closed with sterile tape. After cleaning the sample, muscle will be snap frozen in liquid nitrogen.

Liquid Calorie Diet (800kcal/d)

These diets at this calorie level have previously been used by Dr Heilbronn in overweight subjects for research purposes (with 11/12 patients achieving 15% weight reduction in 8-10 weeks), they are routinely prescribed for obese patients by dieticians and GPs and are a safe and efficient method of weight loss. In this study, participants will be required to lose 10% of their initial body weight and will be monitored bi-weekly during this time. An 800-kcal/d diet with 70g of high quality protein delivered in calorie controlled liquid portions is likely to give optimal weight loss with minimal hunger. Since subjects with a BMI between 25 and 30kg/m^2^ will have a smaller caloric deficit than obese, it may require up to 8 weeks for 10% weight loss.

**Expected Discomfort and Possible risks with the above procedures**

Blood sampling and Clamp- bruising or clotting of the vein can occur but this is unusual and should resolve without medication and should not constitute an ongoing problem. If glucose is not infused at an appropriate rate, a hypoglycaemic (low blood glucose) reaction can occur. However this is extremely unlikely as the object of this study is to monitor blood glucose very frequently and prevent any deviation from normal (5mmol/l).

DEXA- involves the risk associated with small doses of radiation equivalent to 12-hr. exposure to outdoor radiation.

Biopsy- The biopsies carry the risk of bruising, pain, local infections and small scars. About one in 70 will have damage to the nerve or the skin. This goes away within 6-7 weeks and does not affect muscle or joint function

LCD- With any rapid weight loss, there is small risk of developing gallstones. This risk will be minimised by a fat bolus of 10g per day.

**(b)** **If you are doing research on patients, list the procedures/techniques which would not form part of routine clinical management**

1. DEXA

2. Glucose clamps and indirect calorimetry

3. Muscle and fat biopsies

| **2.3** | Will human placental tissue be used? |  |  |
| --- | --- | --- | --- |
|  |  | **No** | **Yes** |

| **2.4** | Will human embryos or fetal tissue be involved? |  |  |
| --- | --- | --- | --- |
|  |  | **No** | **Yes** |

| **2.4** | **Will human embryos or fetal tissue be involved?** |  |  |
| --- | --- | --- | --- |
|  |  | **No** | **Yes** |

| **2.5** | **Will human tissue be collected for culture or any other purpose?** |  |  |
| --- | --- | --- | --- |
|  |  | **No** | **Yes** |

| **2.6** | **Will somatic cell gene therapy be used?** |  |  |
| --- | --- | --- | --- |
|  |  | **No** | **Yes** |

| **2.7** | **Will recombinant DNA techniques be used?** |  |  |  |
| --- | --- | --- | --- | --- |
|  |  | **No** | **Yes** |  |

| **2.8** | **Will toxins, mutagens, teratogens or carcinogens be used?** |  |  |  |
| --- | --- | --- | --- | --- |
|  |  | **No** | **Yes** |  |

| **2.9** | **Will subjects or researchers be exposed to ionizing radiation?** |  |  |  |
| --- | --- | --- | --- | --- |
|  |  | **No** | **Yes** |  |

If you answered **YES**, provide details of the radiation exposure, including a quantitative assessment of the absorbed dose, supported either by dosimetric calculations or by other information.

Subjects will undergo 2 - 3 Dual X-Ray absorbimetry (DEXA) scans. The DEXA scans are necessary to assess body composition changes at baseline, 1-month and following weight loss (if in the applicable group). Each DEXA scan is 5 microseiverts (equivalent to less than 1 day of background radiation and approximately a tenth of a single chest x-ray).

- 1. **Please list any drugs/devices to be used, and their approval status both overseas and in Australia.**

Neutral (Regular) Insulin only (for euglycaemic clamp). This is routinely available on prescription for diabetic subjects in Australia and overseas.

| **2.11** | **Is this research expected to benefit the subjects directly or indirectly?** |  |  |  |
| --- | --- | --- | --- | --- |
|  |  | **No** | **Yes** |  |

If you answered **YES**, provide details.

No, there is no direct benefit to the participant. However, subjects will be provided with information about their health (lipids, insulin, glucose levels). Overweight subjects will also be asked to lose 10% of their initial body weight which will probably reduce lipids and insulin concentration. The subjects will also gain knowledge on their individual energy requirements for weight maintenance and will have access to a dietician during the study.

| **2.12** | **Will the true purpose of the research be concealed from the subject(s)?** |  |  |  |
| --- | --- | --- | --- | --- |
|  |  | **No** | **Yes** |  |

If you answered **YES**, provide details of the concealment and any debriefing.

| **2.13** | **Will the research induce any psychological or physical stress in the subject?** |  |  |  |
| --- | --- | --- | --- | --- |
|  |  | **No** | **Yes** |  |

If you answered **YES**, state what form this stress will take.

The use of cannulae for venous sampling and the clamp study may cause some persons with fear of needles undue stress. We will only include subjects who are comfortable with the procedures as outlined and all will be offered local anaesthetics to minimise possible distress. Subjects who are claustrophobic will be excluded from the study as the placement of the ventilated hood may cause undue stress. Subjects will be also asked to over-eat by 5225kJ/day (1250kcal/day) over their usual weight maintenance requirements. From calculations based on animal data this level of over-consumption will result in 3.8kg of weight gain. We expect that lean individuals will return to baseline weight within 4-weeks. If this process takes longer than 4-weeks, subjects will be provided with additional counselling and access to the study dietician. As part of the study, the overweight group will reduce weight by 10% by liquid calorie diet that is provided by Garvan.

| **2.14** | **Could participation in the research adversely affect the subject(s)?** |  |  |  |
| --- | --- | --- | --- | --- |
|  |  | **No** | **Yes** |  |

If you answered **YES**, what facilities/trained personnel are available to deal with such problems?

There is a slight risk of bruising from the venipuncture sites, and all care will be taken to minimise this outcome. There is also the risk of bruising and possible scarring from the biopsy. About one in 70 will have damage to the nerve or the skin. This goes away within weeks and does not affect muscle or joint function Supportive care will be given to any patient with any bruising or soreness from the procedure. Trained staff will be available during all procedures. Throughout the duration of the clamp study, a nurse will be constantly available and a doctor will be present/readily accessible.

##### SECTION 3: INDEMNITY, COMPENSATION AND POSSIBLE CONFLICT OF INTEREST

| **3.1** | **(a)** | **Will this research be undertaken on behalf of (or at the request of) a pharmaceutical company, or other commercial entity, or any other sponsor?** | **No** | **Yes** |
| --- | --- | --- | --- | --- |

If you answered **YES**, will the sponsor provide support in money or kind? Provide details.

|  | **(b)** | **If you answered YES to (a), will that entity undertake in writing to abide by either the ABPI Clinical Trial Compensation Guidelines or the APMA Guidelines for Injury Resulting from Participation in an Industry–Sponsored Clinical Trial?** | **No** | **Yes** |
| --- | --- | --- | --- | --- |

|  | **(c)** | **If you answered YES to (a), will that entity undertake in writing to indemnify the institution, the IEC(s) and the researchers?** | **No** | **Yes** |
| --- | --- | --- | --- | --- |

|  | **(d)** | If you answered **YES** to **(a)**, **(b)** or **(c)**, does the sponsor hold a current insurance policy to cover this project? If you answered **YES**, provide a certificate of currency. If you answered **NO**, provide details. | **No** | **Yes** |
| --- | --- | --- | --- | --- |

| **3.2** | **Do the researchers have any affiliation with, or financial involvement in, any organisation or entity with direct or indirect interests in the subject matter or materials of this research?** | **No** | **Yes** |
| --- | --- | --- | --- |

If you answered **YES**, provide details.

| **3.3** | **Do the researchers expect to obtain any direct or indirect financial or other benefits from conducting this project?** | **No** | **Yes** |
| --- | --- | --- | --- |

If you answered **YES**, provide details.

| **3.4** | **Are there any further ethical considerations that you wish to raise? For example, have conditions been imposed upon the use, publication or ownership of the results?** | **No** | **Yes** |
| --- | --- | --- | --- |

If you answered **YES**, detail what these considerations are.

**SECTION 4: SUBJECTS**

**4.1 Are the subjects :**

| – 0 - 13 years of age? | **NONE** | **SOME** | **ALL** |
| --- | --- | --- | --- |

| – 14 - 16 years of age? | **NONE** | **SOME** | **ALL** |
| --- | --- | --- | --- |

| – 17 years of age or over? | **NONE** | **SOME** | **ALL** |
| --- | --- | --- | --- |

- 1. **Are the subjects :**

| – unconscious or critically ill patients? | **NONE** | **SOME** | **ALL** |
| --- | --- | --- | --- |
| – mentally ill? | **NONE** | **SOME** | **ALL** |
| – wards of state? | **NONE** | **SOME** | **ALL** |
| – prisoners? | **NONE** | **SOME** | **ALL** |
| – members of the armed services? | **NONE** | **SOME** | **ALL** |
| – in a doctor–patient relationship or a health giver–receiver relationship with the researchers or their associates? | **NONE** | **SOME** | **ALL** |
| – in a teacher–student relationship with the researchers or their associates? | **NONE** | **SOME** | **ALL** |
| – in an employer–employee relationship with the researchers or their associates? | **NONE** | **SOME** | **ALL** |
| – in any other dependent relationship with the researchers or their associates? | **NONE** | **SOME** | **ALL** |

If you answered **YES** to any of the above, provide details.

We will recruit by advertisement on radio and in newspapers. We will not exclude participants from the study if they are in the armed forces or individuals who are employed/students at Garvan or St Vincent’s Hospital should they wish to participate in the study. No employer will be coerced into participating.

- 1. **If the subjects are to undergo a medical or other procedure are they :**

|  | – | capable of understanding the general nature and effects of the proposed treatment? | **No** | **Yes** |
| --- | --- | --- | --- | --- |

|  | – | capable of indicating whether they consent or do not consent to the proposed treatment? | **No** | **Yes** |
| --- | --- | --- | --- | --- |

|  | – | If you answered **NO** to either of the above, is the treatment a new treatment that has not yet gained the support of a substantial number of medical practitioners or dentists specialising in the area of practice concerned? | **No** | **Yes** |
| --- | --- | --- | --- | --- |

|  | – | Has the treatment been declared to be special treatment under the terms of the Guardianship Act 1987? (as amended). | **No** | **Yes** |
| --- | --- | --- | --- | --- |

**SECTION 5: RECRUITMENT OF SUBJECTS**

**5.1 (a) How many subjects will be recruited?**

**40**

1. **How will the subjects be recruited?**

Subjects will be recruited by advertisement in newspapers and on radio

| **5.2** | **(a)** | **Does recruitment involve a direct personal approach from the researchers to the potential subjects?** | **No** | **Yes** |
| --- | --- | --- | --- | --- |

|  |  | If you answered **YES**, is there any pressure from researchers or others that might influence the potential subject to enrol? | **No** | **Yes** |
| --- | --- | --- | --- | --- |

If you answered **YES**, explain.

|  | **(b)** | **Does recruitment involve the circulation/publication of an advertisement, circular, letter, etc?** | **No** | **Yes** |
| --- | --- | --- | --- | --- |

If you answered **YES**, provide a copy and indicate where and how often it will be published.

A copy of the advertisement is included. We plan to utilise the Health Section of the Sydney Morning Herald and will advertise on an as needed basis.

| **5.3** | **Will subjects receive any financial or other benefits as a result of participation?** | **No** | **Yes** |
| --- | --- | --- | --- |

If you answered **YES**, what is the amount/benefit and the justification for this ?

Subjects will receive an amount up to $400 to pay for taxis or other transport costs, childcare or other associated expenditures for attending the Garvan Institute. Subjects will also be provided with their meals for 3-days prior to each metabolic testing day to standardise macronutrient composition, which is necessary for successful completion of the project.

| **5.4** | **Is the research targeting any particular ethnic or community group?** | **No** | **Yes** |
| --- | --- | --- | --- |

If you answered **YES**, which group is being targeted?

|  | If you answered **YES**, has this been done in consultation with a representative of this group? | **No** | **Yes** |
| --- | --- | --- | --- |

If you have not consulted a representative of this group, give reasons.

If you have consulted a representative, who have you consulted and how do they represent this group?

**SECTION 6: PRIVACY AND PUBLICATION OF RESULTS**

**6.1 Is there a requirement for the researchers to obtain information of a personal nature about individuals without their consent :**

|  | – | From Commonwealth departments or agencies? | **No** | **Yes** |
| --- | --- | --- | --- | --- |

|  | – | From other third parties, such as universities, hospitals, State government agencies or employers? | **No** | **Yes** |
| --- | --- | --- | --- | --- |

If you answered **YES**, state what information will be sought and why written consent will not be obtained from the individual subjects.

| **6.2** | **Will any part of the experimental procedures be placed on audio tape, film/video, or other electronic medium?** | **No** | **Yes** |
| --- | --- | --- | --- |

If you answered **YES**, what is the medium and how it will be used ?

| **6.3** | **Is there any possibility that information of a personal nature could be revealed to persons not directly connected with this project?** | **No** | **Yes** |
| --- | --- | --- | --- |

If you answered **YES**, provide details.

**6.4 (a) How will the results of the study be disseminated?**

Results of the study will be analysed in and prepared for publication and/or presentation at scientific meetings. For all presentations of the data, confidentiality will remain strictly enforced.

**(b) How will the confidentiality of data collected/disseminated, including the identity of subjects, be ensured ?**

All personal information collected will be kept on a central database which is password protected and only accessible by CRF staff. Furthermore, only staff involved in the study will have access to case files, which are stored in a locked location. Participants will be identified by participant ID numbers only on blood/biopsy samples.

**(c) What is the proposed storage of, and access to, files, audiotapes etc during the study?**

Storage of experimental data files and personnel data will be kept at the Garvan Institute of Medical Research. The files will remain in a locked secure place until the completion of the study and presentation of results.

**(d) Specify how long the data files/audiotapes will be retained after the study and how they will be disposed of.**

Study files will be stored and retained on the premises of the Garvan Institute of Medical research for 15 years and later stored in archives in a separate location

**SECTION 7: SUBJECT INFORMATION AND CONSENT**

| **7.1** | Will a Subject Information Statement be provided? | **No** | **Yes** |
| --- | --- | --- | --- |

If you answered **NO**, give reasons.

| **7.2** | Will written consent be obtained? | **No** | **Yes** |
| --- | --- | --- | --- |

If you answered **NO**, give reasons.

| **7.3** | **In the case of subjects for whom English is a second language, will arrangements be made to ensure comprehension of the Subject Information Statement and Consent Form?** | **No** | **Yes** |
| --- | --- | --- | --- |

If you answered **NO**, give reasons. If you answered **YES**, what arrangements have been made?

Health Service interpreters will be used for participants or parent/guardian in whom English is their second language

**7.4 (a) Do the Subject Information Statement and Consent Form :**

|  | – | give the title of the project on every page? (Use a short title as appropirate | **No** | **Yes** |
| --- | --- | --- | --- | --- |

|  | – | are the page numbers expressed as page 1 of .., 2 of .., 3 of .. etc? | **No** | **Yes** |
| --- | --- | --- | --- | --- |

|  | – | include an assurance that participation is voluntary and subjects are permitted to withdraw from the project at any time without penalty or prejudice? | **No** | **Yes** |
| --- | --- | --- | --- | --- |

|  | – | include an assurance that participation is voluntary and subjects are permitted to withdraw from the project at any time without penalty or prejudice? | **No** | **Yes** |
| --- | --- | --- | --- | --- |

|  | – | give the name and telephone number of an appropriate investigator? | **No** | **Yes** |
| --- | --- | --- | --- | --- |

|  | – | give a telephone number for an Executive Officer of the IEC, should a subject wish to make a complaint about the conduct of the research project? | **No** | **Yes** |
| --- | --- | --- | --- | --- |

If you answered **NO** to any of the above, give reasons.

|  | **(b)** | **Are the first page of the Subject Information Statement and Consent Form printed on appropriate institutional letterhead?** | **No** | **Yes** |
| --- | --- | --- | --- | --- |

**SECTION 8: DESCRIPTION OF PROJECT**

**8.1 Describe the project in lay terms including the aims, hypotheses, potential significance and research plan (including inclusion/exclusion criteria, where relevant). You must satisfy the IEC that the study is valid and in accordance with accepted principles governing research involving humans. Where relevant, provide the projected number, sex and age range of subjects. The description must be no longer than 2 pages and must be in a font size of at least 10 points.**

The specific aims of this proposal are to:

1. Characterise the physiological, endocrine and anthropometric responses to short-term overfeeding (3d and 4wks) in lean and overweight subjects with and without a genetic predisposition for T2DM.
2. Characterise the physiological, endocrine and anthropometric responses to subsequent weight loss in overweight subjects.
3. Identify mechanisms dysregulating fatty acid oxidation and oxidative phosphorylation in skeletal muscle by measuring fat oxidation in muscle homogenates, as well as by gene expression, protein and enzymatic assays.

**Research Plan:**

**Subjects:**

| **FH-** | 20 lean to overweight subjects with no family history of T2DM |
| --- | --- |
| **FH+** | 20 lean to overweight subjects who have >1 first degree relative with T2DM |

Groups will be matched for sex and age.

Inclusion Criteria:

- Sedentary (<60 min formal exercise per week)
- Aged 20-65 years

Exclusion Criteria:

- Personal history of diabetes, cardiovascular disease, hypertension or eating disorder
- Recent weight change (larger than 3kg in the past 3 months)
- Smoking
- Regular use of medications, except oral contraceptives
- Individuals with alcoholism or other substance abuse
- Pregnancy or lactation, women who are planning to become pregnant or who are not using adequate measures of birth control.

**Screening Visit:** Participants will attend the CRF so that fasting blood samples can be collected (for measurement of glucose and insulin) and resting metabolic rate (RMR) will be determined. Subjects will give a 3-day diet history and will discuss their usual eating patterns with a study dietician. Subjects will then go to SVH for a DEXA scan. The total visit time will be 2-hours.

**Study Design:**

**
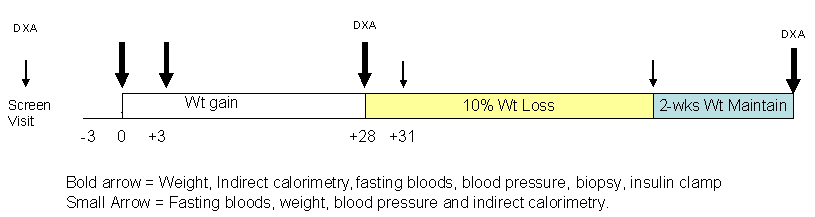
**

Bold arrows denote a long day CRF visit (4-5h) and small arrows indicate a short visit (1-2 h). Three days of meals will be provided to subjects at required energy amounts before each long visit testing day to standardise nutrient composition (eg -3 to +3). Between days +3 and 25 subjects will follow their usual diets but will be provided with high energy snacks, drinks and desserts. These snacks will increase energy intake by 1250kal/day, while minimizing feelings of fullness. Subjects will drink IsoSource 1.5Cal® milkshakes plus BeneCal (500kcal) and will choose from lists of high energy foods that are ~250 kcals per serve (eg: chocolate bars, potato crisps, chocolate mousse). Subjects will be asked to record whether the total menu prescribed was consumed daily and will fill out 3-day diet diaries on two occasions during the study. During the overfeeding phase, participants will attend the CRF once per week to visit the dietician, track weight change and answer a brief activity questionnaire.

Lean participants will only be tested during the overfeeding phase of the study (d28). We expect lean subjects will return to their usual weight within 4-weeks, but they will be offered access to the dietician to assist this process if necessary. Overweight subjects will be placed on liquid calorie diets (800kcal/d, OptiFast, Novartis Nutrition) until 10% weight loss is achieved (6-8weeks). Any subjects not achieving 10% weight loss by 10weeks will be deemed non-compliant and withdrawn from the study. Subjects will return to the clinic for counselling, weight and activity level checks every 2 weeks. Subjects will also return to the clinic at 3-days following initiation of the LCD diet and after 10% weight loss for metabolic testing. Once 10% weight loss is achieved, subjects will be slowly re-fed to weight maintenance over 7-10 days and then be placed back on original meal plan (minus 15% of calories for weight maintenance) for 3-d before the final visit is conducted.

All metabolic tests will be conducted at the Garvan Clinical Research Unit following a 12h overnight fast. Briefly, **weight,** will be measured in a hospital gown after the participant has voided. **Height, blood pressure, waist and hip circumference** will be also measured as previously described (19). Insulin sensitivity will be measured by the gold standard, a **2-h** **hyperinsulinemic euglycaemic clamp** (60mU/m^2^/min). We will also measure **resting metabolic rate** for 30 minutes fasting and during the last 30-minutes of the clamp. Fasting serum samples will be collected and stored at –80°C until study completion for measurement of glucoregulatory matabolites and hormones, lipids and adipocytokines. A muscle and fat biopsy will be performed before the clamp. Following the clamp, lunch will be served. Body composition will be measured by **DXA** at screening, d28 and after weight loss.

##### DECLARATION OF RESEARCHERS

***I/we apply for approval to conduct the research. If approval is granted, it will be undertaken in accordance***

***with this application and other relevant laws, regulations and guidelines.***

**Signature of Chief Investigator or Supervisor**

| Name (print) |  | Signature: |  | Date: |  |
| --- | --- | --- | --- | --- | --- |

**Signature of Associate Investigator(s) or Student(s)**

| Name (print) |  | Signature: |  | Date: |  |
| --- | --- | --- | --- | --- | --- |

| Name (print) |  | Signature: |  | Date: |  |
| --- | --- | --- | --- | --- | --- |

| Name (print) |  | Signature: |  | Date: |  |
| --- | --- | --- | --- | --- | --- |

| Name (print) |  | Signature: |  | Date: |  |
| --- | --- | --- | --- | --- | --- |

***After careful consideration and appropriate consultation, I am satisfied that the scientific merit of***

***this work justifies its being performed and that the information which will be obtained justifies***

***the inconvenience, discomfort and risks to subjects.***

**Signature of appropriate senior officer NOT ASSOCIATED with the research (e.g. Head of School /**

**Department / Unit / Dean of Faculty) .**

| Name (print): |  |
| --- | --- |
| Title (print): |  |
| Position (print): |  |
| Signature: |  |
| Date: |  |

**Acknowledgments.**

The assistance of the following in producing this form is gratefully acknowledged: Mrs Gail Briody (University of Sydney), A/Professor David Cook (University of Sydney/Central Sydney Area Health Service), Professor Simon Gandevia (University of NSW), Dr Rob Loblay (University of Sydney/Australian Health Ethics Committee), Mr Ted McKeown (Hunt & Hunt), Ms Lesley Townsend (Central Sydney Area Health Service), Dr John Watson (University of Sydney), Mrs Margaret Wright (University of NSW).
